# Mechanisms of Cardiometabolic Disparities: Stressor Characteristics Linked to HPA-Axis Dysregulation

**DOI:** 10.1093/geroni/igaa057.1900

**Published:** 2020-12-16

**Authors:** Julie Ober Allen, Briana Mezuk, Tomohiro Ko, Jane Rafferty, Jamie Abelson, Viktoryia Kalesnikava, James Jackson

**Affiliations:** 1 University of Oklahoma, Ann Arbor, Michigan, United States; 2 University of Michigan School of Public Health, Ann Arbor, Michigan, United States; 3 University of Michigan ISR, Ann Arbor, Michigan, United States; 4 University of Michigan, Ann Arbor, Michigan, United States

## Abstract

Diurnal cortisol slopes are stress-sensitive HPA-axis biomarkers implicated in cardiometabolic health outcomes and disparities. This study used two longitudinal cohort studies (CREATE and TRIAD) with harmonized variables to comprehensively examine what types of exposure to stressors are most salient for cortisol dysregulation in later life, and whether the characteristics of stressor exposure accounts for Black-White disparities in cortisol dysregulation (merged sample N=209, 65% male, mean age 61, 17% Black). Black participants reported greater stressor exposure than Whites along some dimensions (e.g., # recent major stressors, appraised severity of lifetime stressors, all p<.02) but comparable exposure in others (e.g., # of lifetime stressors and life domains). Stressor exposure measures that captured psychological components (i.e., appraised severity, psychological distress) and pervasiveness (i.e., # life domains with major stressors) were more closely related to cortisol dysregulation than more objective measures (e.g., # of recent /lifetime stressors). Everyday discrimination was associated with racial disparities.

